# NIAID Workshop Report: Systematic Approaches for ESKAPE Bacteria Antigen Discovery

**DOI:** 10.3390/vaccines13010087

**Published:** 2025-01-18

**Authors:** Inka Sastalla, Keehwan Kwon, Clayton Huntley, Kimberly Taylor, Liliana Brown, Tamika Samuel, Lanling Zou

**Affiliations:** Division of Microbiology and Infectious Diseases, National Institute of Allergy and Infectious Diseases, National Institutes of Health, Rockville, MD 20852, USA; inka.sastalla@nih.gov (I.S.); keehwan.kwon@nih.gov (K.K.); chuntley@niaid.nih.gov (C.H.); kimberly.taylor3@nih.gov (K.T.); liliana.brown@nih.gov (L.B.); tamika.samuel@nih.gov (T.S.)

**Keywords:** ESKAPE bacteria, antigen, vaccine

## Abstract

On 14–15 November 2023, the National Institute of Allergy and Infectious Diseases (NIAID) organized a workshop entitled “Systematic Approaches for ESKAPE Bacteria Antigen Discovery”. The goal of the workshop was to engage scientists from diverse relevant backgrounds to explore novel technologies that can be harnessed to identify and address current roadblocks impeding advances in antigen and vaccine discoveries for the ESKAPE pathogens (*Enterococcus faecium*, *Staphylococcus aureus*, *Klebsiella pneumoniae*, *Acinetobacter baumannii*, *Pseudomonas aeruginosa,* and *Enterobacter* species). The workshop consisted of four sessions that addressed ESKAPE infections, antigen discovery and vaccine efforts, and new technologies including systems immunology and vaccinology approaches. Each session was followed by a panel discussion. In total, there were over 260 in-person and virtual attendees, with high levels of engagement. This report provides a summary of the event and highlights challenges and opportunities in the field of ESKAPE vaccine discovery.

## 1. Current Landscape—ESKAPE Bacterial Pathogens and Vaccines

The 2020–2025 U.S. National Action Plan to Combat Antibiotic Resistant Bacteria established a goal, among others, to accelerate research to develop vaccines that prevent infections with antimicrobial resistant (AMR) bacteria. In response, the U.S. National Institute of Allergy and Infectious Diseases (NIAID) hosted a workshop on 14–15 November 2023 to engage scientists from diverse relevant backgrounds to explore novel technologies that can be harnessed to identify and address current roadblocks impeding advances in antigen and vaccine discoveries for the ESKAPE pathogens (*Enterococcus faecium*, *Staphylococcus aureus*, *Klebsiella pneumoniae*, *Acinetobacter baumannii*, *Pseudomonas aeruginosa* and *Enterobacter* species) (Supplementary Material: Workshop Details S1).

The workshop began with presentations from Drs. Mayora Walters (CDC) and Timothy Cooke (Omniose), who presented information on the public health burden of ESKAPE pathogens, and the status of vaccines in development (Table 1). AMR bacteria pose a worldwide healthcare challenge, and the U.S. Centers for Disease Control and Prevention (CDC) have specifically identified ESKAPE pathogens as urgent threats based on their ability to cause high morbidity and mortality [1,2]. *Staphylococcus aureus*, *Escherichia coli*, *Streptococcus pneumoniae*, *Klebsiella pneumoniae*, and *Pseudomonas aeruginosa* are responsible for approximately 55% of global mortality caused by bacterial infections, with *S. aureus* reported as the leading bacterial cause of death [3]. Most deaths occur in young infants (<1 yr), older adults (>60 yrs), and in hospital settings. In their presentations, Drs. Walters and Cooke discussed how despite numerous efforts over several decades, attempts to combat ESKAPE bacterial infections with vaccines have faced multiple challenges. For example, while each ESKAPE pathogen has its own challenges and limitations, a common theme among all vaccine development efforts is the limited effort in the area of antigen discovery. Overall, ESKAPE vaccine efforts have focused on a limited number of bacterial proteins such as superantigens, adhesins, and iron-scavenging proteins [4,5,6], leading to limited strain coverage or relatively rapid immune escape [7]. Once antigens were down-selected, several development efforts were thwarted due to low vaccine efficacy and safety issues that caused early termination of clinical trials [8].

Dr. Walters described how healthcare-associated infections (HAIs) in the USA are monitored by the National Healthcare Safety Network (NHSN), managed by the CDC. Reported HAIs include central line-associated bloodstream infections (CLABSIs), catheter-associated urinary tract infections (CAUTIs), surgical site infections (SSIs), and selected ventilator-associated events (VAEs). Data collected from this surveillance network are used to track emerging AMR pathogens in the US. For example, thirty eight percent of HAIs reported in the USA were associated with ESKAPE pathogens [10]. Importantly, an ESKAPE pathogen was isolated in more than 60% of cases of ventilator-associated pneumonia (VAP). *S. aureus* was responsible for the majority of VAP, CLABSI, and SSIs, while *Klebsiella* species caused the majority of catheter-associated UTIs. Between 2017 and 2019, decreasing numbers of infections were noted with MDR- *P. aeruginosa* and carbapenem-resistant Enterobacterales (CRE), while carbapenem-resistant *Acinetobacter* (CRA), vancomycin-resistant *Enterococci* (VRE) and methicillin-resistant *S. aureus* (MRSA) showed a stabilizing trend in the number of HAIs. Unfortunately, the decreasing rate of AMR ESKAPE infections was reversed during the COVID-19 pandemic [11]. In addition to the toll on human morbidity and mortality, HAIs were also associated with increased healthcare costs, with MRSA and CRA infections showing the highest attributable costs. As with hospital-acquired infections, ESKAPE pathogens also featured prominently in community-acquired infections, with *E. coli* and *S. aureus* being the leading causes that resulted in sepsis [12]. Altogether, these infections added USD 4.6 billion in US healthcare costs annually [13].

## 2. Challenges and Opportunities

The remainder of the workshop was dedicated to identifying challenges impeding antigen discovery and vaccine development and determining growing opportunities to address these challenges.

### 2.1. Large Variability in Clinical Presentation

The variability in clinical presentations of ESKAPE infections poses challenges in selecting a vaccine-target population critical for developing realistic vaccine approaches. ESKAPE organisms can infect multiple body sites resulting in different clinical symptoms (e.g., *S. aureus* causes soft-tissue infections, pneumonia, or blood-stream infections); can be asymptomatic, mild, severe, or fatal; or may be persistent or recurring [14,15,16,17]. In most cases, it is not known whether vaccines targeting antigens expressed during one clinical presentation can broadly protect against other presentations. For example, vaccines that are based on antigens expressed during bloodstream or tissue infections may not be effective against osteomyelitis caused by the same pathogen [18,19]. In practice, this may require multiple antigens to be identified and included in vaccine formulations to cover the variety of anatomical infection sites, or targeted vaccines may be required that are limited to distinct clinical presentations.

### 2.2. Complex Pathogen Biology

ESKAPE bacteria express a myriad of colonization, virulence, and immune evasion factors at different times and at different body sites during infection. In most cases, ESKAPE bacteria carry multiple genes with redundant functions that could come into play when immune pressure is experienced at one gene [4]. Additionally, horizontal gene transfer and often a high mutational rate of immune-exposed proteins allow these bacteria to acquire new or modify existing virulence factors, thus making vaccine approaches that focus on one or few antigens less likely to succeed [20].

### 2.3. Healthcare-Associated Infections

Most life-threatening infections occur during patient hospitalization, and to reduce the risk of HAIs, prophylactic vaccine interventions are desirable [8,10]. However, effective immunizations need to be administered weeks before the patient’s admission, thus limiting feasibility beyond elective procedures [21]. Short-term treatments such as prophylactic monoclonal antibody regimens might offer a viable alternative.

### 2.4. Limited Information in Human Immune Responses

**Figure 1:**
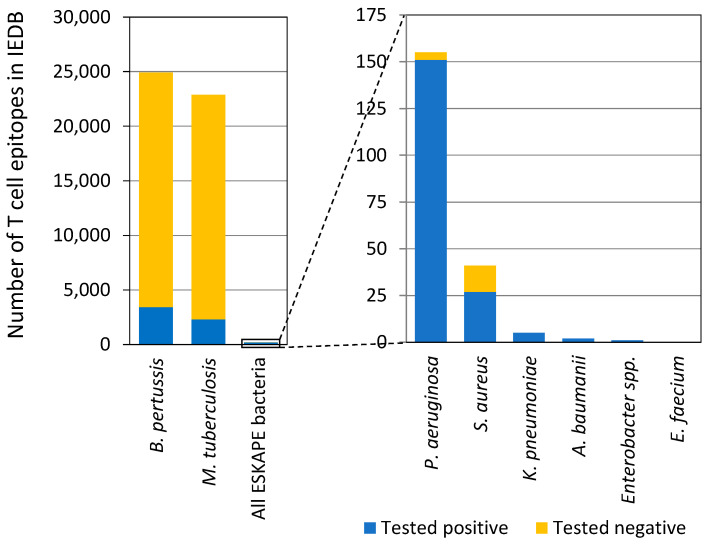
T cell epitope data for ESKAPE bacteria in IEDB. Tested positive/negative indicates T cell assay outcome. This figure is adapted from Dr. Bjoern Peters’ presentation in the workshop.

Past vaccine research predominantly focused on antibodies and B cell responses that target a limited number of bacterial antigens. However, T cell responses to ESKAPE bacteria have been understudied. For example, the Immune-Epitope Database (IEDB) lacks abundant T cell epitope data for all ESKAPE bacteria (Figure 1); in fact, ESKAPE bacteria make up less than 0.3% of bacterial T cell epitope data in IEDB, and past epitope mapping studies have focused on very few selected protein targets, constraining T cell-focused vaccine strategies. Researchers are also exploring the possibility that early-life colonization by ESKAPE bacteria may create a humoral imprint, affecting human immune status and potentially rendering developed vaccines ineffective or even harmful.

### 2.5. Non-Clinically Relevant Vaccine Candidates

Many ESKAPE vaccine candidates show promise in laboratory and animal infection models, but their efficacies often fail to translate to human infection [22]. Better and alternative infection models, with a clearer understanding of correlates of protection, are needed to assist in vaccine development.

### 2.6. Limited Access to Biospecimens

There are existing panels of ESKAPE bacterial strains made available by NIAID, CDC, and FDA. However, there are limited publicly available well-maintained biobanks for human sera and tissue samples, as well as bacterial isolates from longitudinal ESKAPE clinical cases that include detailed metadata. Similarly, there are no agreed-upon criteria to regularly update panels to better represent circulating ESKAPE bacterial strains, and therefore even the existing strain panels might be outdated by the time they are used for vaccine testing. Such reagents and resources would enable the identification of immune correlates associated with infection and greatly benefit the research community.

### 2.7. Available Cutting-Edge Technologies

New and emerging technologies are mostly applied to viral research and are not always applied widely to advance antigen discovery and resolve obstacles that hinder ESKAPE vaccine development.

## 3. Emerging Technologies for Antigen Discovery and Vaccine Development

Emerging technologies can be harnessed to identify ESKAPE antigens. Similarly, new vaccine platforms and approaches can help overcome challenges and populate the ESKAPE bacteria vaccine pipeline. Some promising technologies that are not yet systematically employed for ESKAPE bacteria were discussed.

### 3.1. Host-Bacterium Multi-Dimensional Profiling for Antigen Discovery

Multi-dimensional host-bacterium profiles and bacterial gene interaction screens can support antigen discovery. This technology uses genome-wide bacterial gene deletion screens combined with comparative genomics and transcriptomics to investigate gene interactions and their impact on bacterial behavior and phenotypes under diverse conditions, such as exposure to drugs, different carbon sources, ions, host cells, and in vivo environments. Such approaches can reveal essential bacterial pathways and mechanisms, aid our understanding of bacterial biology contributing to antibiotic resistance, pathogenicity, and environmental adaptation, and may lead to the identification of new vaccine targets [23,24,25].

### 3.2. Reverse Vaccinology

Reverse vaccinology was first described in the early 2000s [26]. Instead of the traditional approach of isolating and growing pathogens in the lab, reverse vaccinology starts with the pathogen’s genetic sequence to predict which proteins might be suitable vaccine targets, thus allowing researchers to prioritize antigen characterization and eliminating lengthy growth and isolation methods for sub-optimal candidates. This approach has been successfully used for the discovery and development of GSK’s *Neisseria meningitidis* serogroup B (MenB) and can further be explored for ESKAPE pathogens [27,28,29,30,31,32].

### 3.3. Systems Immunology and Serology

Technologies such as IVIAT (in vivo induced antigen technology) identify bacterial immunogenic antigens expressed during human infection. Advanced “systems” approaches can combine immune genomics, transcriptomics, and metabolomics to comprehensively analyze both innate and adaptive human host responses during infections. Such systems immunology approaches can help identify correlates of infection and protection and ultimately new antigens for ESKAPE bacteria. Large-scale datasets from human and pathogen during infections allow the systematic and wide-ranging analysis of antibody responses to infection and/or vaccination. Systems serology combines traditional serology techniques with high-throughput technology to comprehensively study the characteristics and dynamics of antibodies. This approach, described at the workshop in the context of human Shigella challenge studies, can provide insights into immune system interactions, disease progression, and vaccine efficacy.

### 3.4. Mass Spectrometry-Based Immunopeptidomics for Antigen Discovery

Immunopeptidomics is a powerful approach to discover novel bacterial antigens. This technology aims to identify and characterize pathogen peptides that are presented by major histocompatibility complex (MHC) molecules. The method employs mass spectrometry and bioinformatics techniques to analyze the repertoire of peptides presented by MHC molecules, thus providing insights into the antigen presentation and immune surveillance mechanisms [33,34]. Additional computational tools for the design of epitope-driven vaccines such as the Vaxign 2 vaccine design platform [35], a machine learning-based vaccine candidate prediction and analysis system, presented at the workshop can evaluate the identified vaccine candidates for *Listeria monocytogenes* [34].

### 3.5. Genome-Wide Peptide-Guided T Cell Epitope Discovery

Peptide-guided T cell epitope discovery first predicts surface epitopes and then systematically screens peptide pools and peptides for their ability to bind to MHC molecules and to activate T cells [36]. This approach has been used to systematically identify T cell epitopes for viruses and—more limited—for bacteria with a focus on genome-wide *M. tuberculosis* and *B. pertussis* epitopes.

### 3.6. Computational Protein Structure Prediction and Modeling

Three-dimensional protein structures are critical to understand biological systems and functions of many uncharacterized ESKAPE proteins, and they can aid antigen discovery and vaccine development. The structural biology field has seen an exponential increase in resources and tools for computational 3D prediction and modeling [37]. In the artificial intelligence (AI) system, AlphaFold 2 revolutionized protein modeling with an accuracy of 94–98% in its predictions. Currently, more than 2 million predicted protein structure models are accessible to the public through the AlphaFold Database. The recent AlphaFold 3 has demonstrated much higher accuracy in predicting complex structures, including proteins with ligands, nucleic acids, antibodies, and other proteins [38,39,40]. Multiple high accuracy computational tools have been developed and evolved for structure-based antigen discovery, designing antigens and antibodies, and for modeling protein complexes with small and macromolecules; tools include “deep network hallucination”, AlphaFold3, RoseTTAFold and Protein MPNN [38,41,42,43,44]. Applications for these tools provide resources for antigen discovery, increasing the breadth of antibodies, and the design for epitope-focused and self-assembly nanoparticle vaccines [43,45,46,47,48].

### 3.7. Computational Tools for T Cell Epitope-Based Vaccines

In vaccine design, the T cell response is one of the critical factors since T cell activation facilitates B cell activation [49,50,51]. A series of immunoinformatics tools, such as iVAX, an integrated toolkit, is available for computational vaccine design from genome sequencing data by identifying antigens and predicting T cell epitopes, epitope density, and immunogenic potential [52,53]). The concept and workflow have been validated in retrospective and prospective studies with influenza virus, *Mycobacterium tuberculosis*, *Burkholderia mallei* and *pseudomallei*, and *Coxiella burnetii* [52].

### 3.8. Vaccine Platforms and Technologies

Current vaccine platforms include recombinant DNA and proteins, outer membrane vesicles (OMVs), glycoconjugates, viral vectors, and nucleic acid (DNA/RNA) technologies [54,55,56]. Several promising new platforms have been discussed that could prove suitable for ESKAPE vaccines. For example, the versatile GSK GMMA platform relies on outer membrane vehicles (OMVs) produced by engineered *E. coli* [55,57,58]. OMVs can include multiple antigens in their natural conformation, provide self-adjuvating activity, and have a high yielding manufacturing process at a relatively low cost. In contrast, glycoconjugation technologies rely on bacterial polysaccharides conjugated to a carrier protein, and more easily manufactured alternatives, such as bioconjugated and multiple antigen-presenting systems (MAPSs), were discussed, some of which have already been tested and developed for select ESKAPE pathogens [54,59,60,61].

## 4. Concluding Remarks

During the workshop, valuable opportunities were identified to advance ESKAPE antigen discovery, develop medical countermeasures, and close scientific and technological gaps. First, research into longitudinal cohort studies and associated biobanks for select human bacterial infections is essential. These resources could be made available to the scientific community to enhance our understanding of pathogenesis, human host immune responses, treatment, and clinical outcomes. Second, utilizing samples and information from longitudinal cohorts could significantly expand human system immunology and system vaccinology studies aimed at identifying molecular signatures, immune markers, and correlates associated with clinical outcomes. Third, research to identify both T cell and B cell antigens and epitopes for ESKAPE bacteria is critical for the development of effective vaccines, which can elicit a robust and long-lasting immune response and help to overcome bacterial evasion strategies. Lastly, the integration of emerging technologies and approaches, such as systems biology and multi-scale methods incorporating computational biology, artificial intelligence, and predictive techniques, can be a pivotal moment to advance the discovery of ESKAPE bacterial epitopes and antigens. These advancements and newly identified antigens and epitopes could lay the foundation for the future development of vaccines, therapeutics, and diagnostics, leveraging newly identified antigens and epitopes.

## Figures and Tables

**Table 1:** Number of ESKAPE vaccine candidates under global (pre-) clinical development in 2021 [8,9]. Two Staphylococcus aureus vaccine candidates are rTSST-1 variant vaccine (Phase 2) and GSK3878858A (Phase 1/2), and a Klebsiella pneumoniae vaccine candidate is KlebV4 (Phase 1/2).

Vaccine Candidates	*Enterococcus faecium* “E”	*Staphylococcus aureus* “S”	*Klebsiella pneumoniae* “K”	*Acinetobacter baumannii* “A”	*Pseudomonas aeruginosa* “P”	*Enterobacter* *ssp.* “E”
Pre-clinical	0	14	5	5	4	0
Clinical	0	2	1	0	0	0

## Data Availability

This report summarizes the workshop, unlike a research or review article, no data was generated, and no software source code was developed. Detailed information about the workshop is provided in the Supplementary Materials.

## Supplementary Materials

The following supporting information can be downloaded at https://www.mdpi.com/article/10.3390/vaccines13010087/s1: Workshop Details S1: The list of speakers, panelists, and moderators.
