# Supplementary material for: NIAID Workshop Report: Systematic Approaches for ESKAPE Bacteria Antigen Discovery
Source: Vaccines (Basel). 2025 Jan 18;13(1):87. doi: 10.3390/vaccines13010087 (PMC11768834; doi:10.3390/vaccines13010087)
Supplement: Supplementary file 1 [file vaccines-13-00087-s001.zip › vaccines-3394495-supplementary.pdf]

NIAID Workshop: Systematic Approaches for ESKAPE Bacteria Antigen Discovery

November 14-15, 2023

NIAID Conference Center – Garden Room 1, 5601 Fishers Lane, Rockville, MD 20892

Sponsored by Division of Microbiology and Infectious Diseases (DMID),
National Institute of Allergy and Infectious Diseases (NIAID)

Purpose: The purpose of this two-day workshop is to foster collaboration between experts with expertise in omics, bacterial infectious diseases research, microbiology, immunology, and vaccinology. Together, they will explore innovative ideas and techniques for systematically identifying ESKAPE pathogen antigens using advanced technologies and integrated tools.

Aims:

- To identify and discuss current challenges in vaccine development for AMR ESKAPE pathogens.
- To explore innovative new ideas, approaches, and technologies for advancing AMR ESKAPE antigen discovery.
- To provide a multi-disciplinary environment with experts in infectious diseases, vaccinology, and multi-omics approaches to identify gaps, challenges, and strategies to accelerate AMR ESKAPE antigen discovery.

Day 1

Keynote Lecture 1

Timothy Cooke (Omniose): Still No ESKAPE: Vaccine and Antibody Development.

Maroya Walters (CDC): Too Much ESKAPE: Epidemiology.

Session 1: ESKAPE Infections, and Vaccine Efforts

Moderator: *Maroya Walters*

Vance Fowler (Duke University)

Experiments of Nature: Using Clinical Biorepositories to Interrogate Systems Immunology in Common Bacterial Infections.

Mariagrazia Pizza (Imperial College London)

Vaccine Discovery and Development Based on Reverse Vaccinology: Past, Present and Future.

Tim van Opijnen (The Broad Institute)

Multidimensional Host-Bacterium Profiles to Guide the Design of New Antimicrobial Strategies.

Siobhán McClean (University College Dublin)

Exploiting Host-Pathogen Interactions – A Proteomic Approach to Identify Effective Vaccine Candidates for ESKAPE Pathogens.

Alexei Savchenko (University of Calgary)

Discovering Novel Antigen Candidates on The Surface of a Gram-Negative Pathogen; Structural Modelling Approach.

<u>Panel Discussion</u>: Vance Fowler, Mariagrazia Pizza, Tim van Opijnen, Siobhán McClean, Alexi Savchenko.

Session 2: New Approaches for Vaccine Antigen Design

Moderator: *C. Buddy Creech* (Vanderbilt University)

M. Javad Aman (AbVacc) and *David Talan* (University of California, Los Angeles)

Informing Vaccine Design and Development by Clinical Epidemiology; Making the Case for a Fully Toxoid *S. aureus* Vaccine.

C. Buddy Creech

Returning the Horse to the Front of the Cart: How Successful Navigation of Infection Can Drive Vaccine Development.

Sara Roggensack (GlaxoSmithKline)

Vaccine Technologies to Combat AMR Pathogens.

Richard Malley (Harvard University)

A MAPS Vaccine to Target *Klebsiella pneumoniae* and *Pseudomonas aeruginosa.*

<u>Panel Discussion</u>: M. Javad Aman, David Talan, C. Buddy Creech, Sara Roggensack; Richard Malley, Nadine Rouphael (Emory University), Christopher Montgomery (The Ohio State University).

Day 2

Keynote Lecture 2

Bali Pulendran (Stanford University): Systems Vaccinology.

Session 3: Human Immune Correlates and ESKAPE Infections

Moderator: *George Y. Liu* (University of California, San Diego)

George Y. Liu

Immune Imprint as an Explanation for the Failure of Staphylococcal Vaccines.

Gregg Silverman (New York University)

Towards an Integrated Approach to Develop Multi-Component Vaccines using Phage Display.

Juliane Bubeck-Wardenburg (Washington University)

Role of Antigen Specificity in Eliciting Protective Immunity against *S. aureus*.

Galit Alter (Moderna)

New Tools to Dissect Protective Antibody Responses to Bacteria.

Panel Discussion: George Y. Liu, Gregg Silverman, Juliane Bubeck-Wardenburg, Galit Alter, Nadine Rouphael, Christopher Montgomery.

Session 4: Systems Immunology Approaches for Antigen Discovery

Moderator: *Bret Sellman* (AstraZeneca)

Francis Impens (Ghent University, Belgium)
Immunopeptidomics for Next-Generation Bacterial Vaccine Development.

Bjoern Peters (La Jolla Institute for immunology)
T Cell Epitope Discovery in Bacteria: Current Status and Challenges.

Kirk Haltaufderhyde (EpiVax Inc)
iVAX: A Platform for Epitope Driven Vaccine Designs.

Jens Meiler (University Leipzig, Germany & Vanderbilt University)
Computational Approaches to Engineering Antibodies and Vaccines.

Panel Discussion: Francis Impens, Bjoern Peters, Kirk Haltaufderhyde, Jens Meiler.
